# Differential Host Gene Expression in Response to Infection by Different *Mycobacterium tuberculosis* Strains—A Pilot Study

**DOI:** 10.3390/microorganisms12112146

**Published:** 2024-10-25

**Authors:** Dewi Megawati, Lisa Y. Armitige, Loubna Tazi

**Affiliations:** 1 Department of Medical Microbiology and Immunology, School of Medicine, University of California, Davis, CA 95616, USA; amegawati@ucdavis.edu; 2 Department of Microbiology and Parasitology, Faculty of Medicine and Health Sciences, Warmadewa University, Denpasar 80239, Bali, Indonesia; 3 The Heartland National TB Center, San Antonio, TX 78223, USA; lisa.armitige@dshs.texas.gov

**Keywords:** *Mycobacterium tuberculosis*, expression profiling genes, antimicrobial resistance, interferon-stimulated genes (ISGs), host–pathogen interaction

## Abstract

Tuberculosis (TB) represents a global public health threat and is a leading cause of morbidity and mortality worldwide. Effective control of TB is complicated with the emergence of multidrug resistance. Yet, there is a fundamental gap in understanding the complex and dynamic interactions between different *Mycobacterium tuberculosis* strains and the host. In this pilot study, we investigated the host immune response to different *M. tuberculosis* strains, including drug-sensitive avirulent or virulent, and rifampin-resistant or isoniazid-resistant virulent strains in human THP-1 cells. We identified major differences in the gene expression profiles in response to infection with these strains. The expression of IDO1 and IL-1β in the infected cells was stronger in all virulent *M. tuberculosis* strains. The most striking result was the overexpression of many interferon-stimulated genes (ISGs) in cells infected with the isoniazid-resistant strain, compared to the rifampin-resistant and the drug-sensitive strains. Our data indicate that infection with the isoniazid-resistant *M. tuberculosis* strain preferentially resulted in cGAS-STING/STAT1 activation, which induced a characteristic host immune response. These findings reveal complex gene signatures and a dynamic variation in the immune response to infection by different *M. tuberculosis* strains.

## 1. Introduction

Tuberculosis (TB), caused by *Mycobacterium tuberculosis* infection, represents a disease of global public health importance and remains a leading cause of morbidity and mortality. It is the second most common cause of death due to a single infectious agent after COVID-19, surpassing HIV/AIDS [1,2]. About a quarter of the world’s population is currently infected with *M. tuberculosis*, with more than 10 million new cases of active TB reported globally, resulting in more than 1.5 million deaths annually. The emergence of drug-resistant TB and the increasing incidence of multidrug-resistant (MDR) and, more recently, of extensively drug-resistant (XDR) and totally drug-resistant (TDR) *M. tuberculosis* strains are an important public health threat and jeopardize current efforts to TB control and prevention [3,4,5,6]. About 450,000 cases of MDR-TB are recorded each year, with a global treatment success rate of 60% [1]. Resistance to rifampin and/or isoniazid is of major concern, since these two drugs represent the cornerstone of the first-line TB treatment, and resistance to both drugs is linked to all multidrug-resistant forms of TB (MDR, XDR, and TDR). Furthermore, monoresistance to isoniazid is noticeably common in contrast to monoresistance to rifampin. Resistance to rifampin typically occurs in strains that are also resistant to isoniazid. While rifampin is unequivocally the most important drug in the global fight against active TB, isoniazid is critical not only for the management of active TB, but it has long been the standard of care for latent TB treatment [7].

The *M. tuberculosis* cell wall has a complex composition and structure. It is considered to be a major virulence factor and to promote the resistance of *M. tuberculosis* to antibiotics [8]. Interestingly, the cell wall is significantly thicker in drug-resistant *M. tuberculosis* strains compared to drug-sensitive strains [9,10]. Cell wall lipids in *M. tuberculosis* play a significant role in modulating the host immune response, pathogenesis, and virulence [11,12,13,14]. Mycolic acids represent the hallmark of the *M. tuberculosis* cell wall, and their biosynthesis and regulation are the targets of isoniazid [15,16]. Upon exposure to isoniazid, *M. tuberculosis* uses different tolerance strategies to overcome the inhibition of mycolic acids biosynthesis induced by isoniazid [17]. As the concentration of mature mycolic acids decreases, the lipid composition becomes altered, which influences *M. tuberculosis* physiology and host pathogenesis [14,18]. Significant changes in lipid metabolism (β-oxidation of fatty acids) after the acquisition of isoniazid resistance have also been documented [19,20]. While isoniazid blocks the synthesis of the cell-wall mycolic acids, the mechanism of action of the other major anti-TB drug, rifampin, is to inhibit mycobacterial transcription by targeting DNA-dependent RNA polymerase. Resistance to rifampin has been mainly linked to mutations in the gene encoding the β-subunit of RNA polymerase (*rpoB* gene) [21].

*M. tuberculosis* is remarkably successful at evading the host immune response [22]. This pathogen adopts several strategies to avoid clearance and persist in the host [23,24]. Macrophages play a crucial role in host defense against *M. tuberculosis* by promoting the activation of different signaling pathways [25,26]. In particular, cGAS, TBK1, and NF-κB are known to be strongly involved in the activation of the innate immunity by the release of many cytokines, such as IL-1β, and the expression of many interferon-stimulated genes (ISGs) [27,28]. Nevertheless, our understanding of the host–*M. tuberculosis* interactions is still limited, and many fundamental gaps in our knowledge remain in how these interactions may differ in response to distinct *M. tuberculosis* strains [29,30].

Infections with different *M. tuberculosis* genotypes have major impacts on host–pathogen interaction, which can lead to substantial differences in the host immune responses [31,32,33]. The emergence of some mycobacterial lineages, such as the Beijing genotype family, has even been linked to an evolutionary adaptive response to drug resistance [34]. Beijing strains have shown specific properties in their interaction with the host immune system, suggesting a possible human–pathogen co-evolution. The immune response of infected macrophages also showed a wide variation in the response to different *M. tuberculosis* strains [35]. All of these studies contributed with others to substantial advances in our understanding of TB pathogenesis. However, further work is needed to explore the host immune response to different *M. tuberculosis* strains and how these differences in immune responses could affect TB pathogenesis. In this pilot study, we tackled this question and investigated the host immune response to different reference *M. tuberculosis* strains, including drug-sensitive avirulent or virulent, and rifampin-resistant or isoniazid-resistant virulent strains in THP-1 cells. Shifting the focus to the specific impact on differential host interaction with *M. tuberculosis* strains has the potential to improve our understanding of TB pathogenesis and initiate efforts for a roadmap towards better TB control and prevention strategies.

## 2. Materials and Methods

### 2.1. Cell Line and M. tuberculosis Strains

For this study, we used human monocytic THP-1 cells (American Type Culture Collection; Catalog No. TIB-202). The cells were maintained in RPMI 1640 medium supplemented with 10% heat-inactivated fetal bovine serum (FBS) at 37 °C and 5% CO_2_ in a humidified incubator. THP-1 cells were subsequently differentiated for 48 h with 10 ng/mL phorbol 12-myristate 13-acetate (PMA). The cells were then infected with four different reference mycobacterial strains (H37Ra, H37Rv, H37Rv-INH-R, and H37Rv-RIF-R) at an MOI of 10, as commonly used in other studies [36,37,38]. Both H37Ra (avirulent strain; American Type Culture Collection; Product No. 25177) and H37Rv (virulent strain; American Type Culture Collection; Product No. 27294) are drug-sensitive. H37Rv-INH-R (American Type Culture Collection; Product No. 35822) and H37Rv-RIF-R (American Type Culture Collection; Product No. 35838), are both derivatives of H37Rv, and they are resistant to isoniazid and rifampin, respectively. We used two time points for infection (4 and 24 h) in order to monitor differences in gene expression patterns for differentially expressed genes. In addition to the infected THP-1 samples, we also used uninfected control cells (mock-infected with PBS) as negative controls.

### 2.2. RNA Isolation and Processing

Total RNA was extracted from controls and infected cells, using TRIzol (Invitrogen, Carlsbad, CA, USA) according to the manufacturer’s protocol. Ten samples were used in total, with 5 samples (negative control, H37Ra, H37Rv, H37Rv-INH-R, and H37Rv-RIF-R) at 4 h and 24 h, representing early infection and late infection, respectively [39]. RNA was then suspended in RNase-free water and stored at −80 °C until further use. RNA quality was assessed to verify its integrity using an Agilent 2100 Bioanalyzer (Agilent Technologies, Santa Clara, CA, USA), and RNA quantity was evaluated by spectrophotometry using NanoDrop 2000 (NanoDrop Technologies, Wilmington, DE, USA). All RNA samples showed a good RNA yield and no RNA degradation. Total RNA was then reverse transcribed to cDNA, amplified, labeled, and hybridized to separate arrays using the GeneChip Human Exon 1.0 ST Array (Affymetrix, Santa Clara, CA, USA), according to the manufacturer’s instructions. For transcriptional profiling, we used triplicate hybridization assays in our microarray experiments in order to assess variability among independent labeling reactions and hybridizations, as well as variation for gene expression in the infected cells [40]. Quality control of the hybridized arrays was also performed for each sample. A visual inspection of the scanned images was conducted, looking for any defects, areas of high background, or areas of low signal. The spike-in controls were checked as well to examine for hybridization uniformity.

### 2.3. Microarray Analysis for Differential Gene Expression

Data from all 10 samples were preprocessed and then summarized at the transcript-cluster (gene) level, and RMA was normalized using Affymetrix Power Tools. Prior to differential expression analysis, low-variability genes were filtered out, leaving 13,460 genes. Differentially expressed genes (DEGs) were identified using the R/Bioconductor package *limma* [41]. Significant differential expression was defined by a false discovery rate (FDR)-adjusted *p*-value of less than 0.05, which is calculated by the Benjamini–Hochberg procedure [42]. The visualization of the DEGs in the volcano plots was performed using base R graphics. The R statistical software (version 3.3.3) was also used to produce a heatmap based on the top 100 most significant DEGs in THP-1 cells infected with H37Rv-INH-R relative to mock-infected cells at 24 h post-infection. In order to identify the overlapping DEGs in our data, we generated Venn diagrams, as well using VennDiagram package in R [43,44]. The complete dataset for the microarray analysis of all 10 samples was deposited in the NCBI Gene Expression Omnibus database (GEO; https://www.ncbi.nlm.nih.gov/geo/; accessed on 22 October 2024) and can be publicly accessed through GEO Series accession number GSE246736.

### 2.4. Functional Enrichment Analysis

To categorize the biological functions of the DEGs in our dataset, we used the DAVID database (https://david.ncifcrf.gov, accessed on 22 October 2024) to perform a Gene Ontology (GO) term enrichment analysis of biological processes, cellular components, and molecular functions; and a Kyoto Encyclopedia of Genes and Genome (KEGG) pathway enrichment analysis [45,46]. A *p*-value < 0.05 was used as the cut-off value for screening significantly enriched DEGs.

## 3. Results

### 3.1. Identification of Differentially Expressed Genes

**Figure 1:**
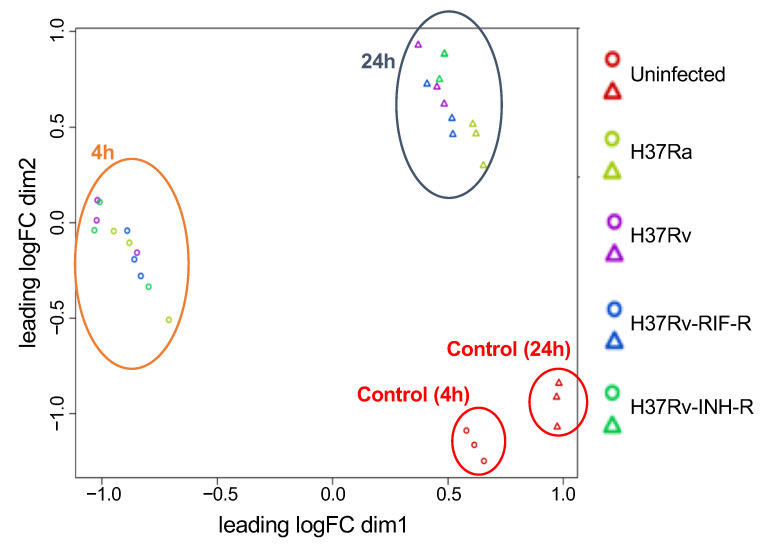
Multidimensional scaling (MDS) plot based on the expression of all microarray genes between *M. tuberculosis*-infected and mock-infected THP-1 cells at two time points of infection. Circles represent data based on 4 h post-infection, and triangles depict data based on 24 h post-infection. Each specifically colored circle or triangle represents a replicate of each sample.

In this pilot study, we generated gene expression profiles of THP-1 cell lines infected with the following *M. tuberculosis* reference strains: H37Ra, H37Rv, H37Rv-INH-R, and H37Rv-RIF-R. We chose to use THP-1 cells because they have been shown to display comparable mycobacterial uptake, cellular viability, and cytokine/chemokine induction profiles as human monocyte-derived macrophages [47,48]. Previous studies have shown different transcriptomic profiles comparing THP-1 cells infected with H37Rv, H37Ra, and BCG vaccine [49,50]. These cells can also be reliably grown in the lab and allow us to avoid the putative confounding effects that might arise from genetic differences within macrophages isolated from different donors. The multidimensional scaling (MDS) plot for the microarray expression data showed well-separated grouping according to infections vs. mock infections, and time points of infections (Figure 1).

**Figure 2:**
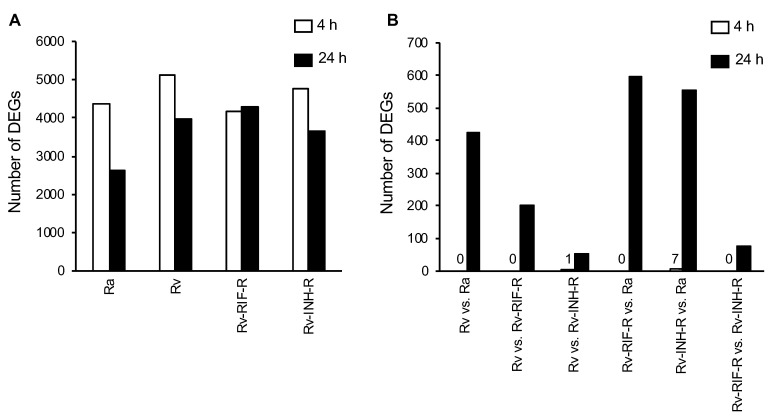
Number of differentially expressed genes (DEGs) in THP-1 cells at 4 h and 24 h post-infection (*p* < 0.05). (**A**) *M. tuberculosis*-infected cells relative to mock-infected cells. (**B**) Pairwise comparisons of *M. tuberculosis*-infected cells.

A large number of DEGs were detected between infected and mock-infected cells, with the virulent *M. tuberculosis* strains (H37Rv, H37Rv-INH-R, and H37Rv-RIF-R) showing more DEGs than the non-virulent strain (H37Ra) at 24 h post-infection (Figure 2A). Pairwise comparisons between cells infected with the different strains showed only a few DEGs at 4 h post-infection, but higher numbers after 24 h of infection, with most DEGs detected when avirulent and virulent strains were compared (Figure 2B). Our comparison of H37Rv- with H37Rv-INH-R- and H37Rv-RIF-R-infected cells revealed 55 and 201 DEGs 24 h post-infection, respectively. Based on these findings, the subsequent analyses were therefore focused on the 24-h time point.

**Figure 3:**
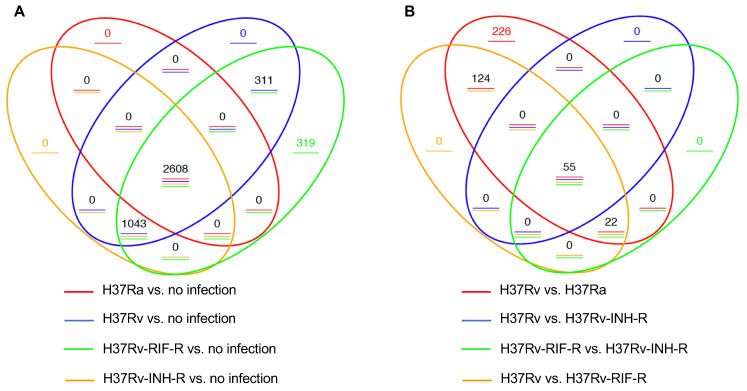
Venn diagrams of DEGs in THP-1 cells at 24 h post-infection (*p* < 0.05). The overlapping genes represent the overall number of DEGs between the different comparison groups, while the non-overlapping numbers designate the unique genes to each group. (**A**) Pairwise comparisons between *M. tuberculosis*-infected and mock-infected cells. (**B**) Pairwise comparisons between *M. tuberculosis*-infected cells.

We next generated Venn diagrams to identify overlapping DEGs. We first analyzed the DEGs by comparing infected and non-infected THP-1 cells. A total of 2608 DEGs were shared between all comparison groups (Figure 3A). In total, 1043 DEGs were shared in the comparison with cells infected with the virulent strains (H37Rv, H37Rv-INH-R, and H37Rv-RIF-R), but not in the group with H37Ra-infected cells. Of note, 319 DEGs stood for a unique profile associated with H37Rv-RIF-R-infected cells. The pairwise comparisons between infected cells showed a total of 55 common DEGs, which represent the overall number of DEGs significant in all four comparisons, with 226 unique genes which were only significant in the pairwise-comparison group H37Rv- vs. H37Ra-infected THP-1 cells (Figure 3B).

### 3.2. Differential Gene Expression in Isoniazid-Resistant M. tuberculosis Infection

**Figure 4:**
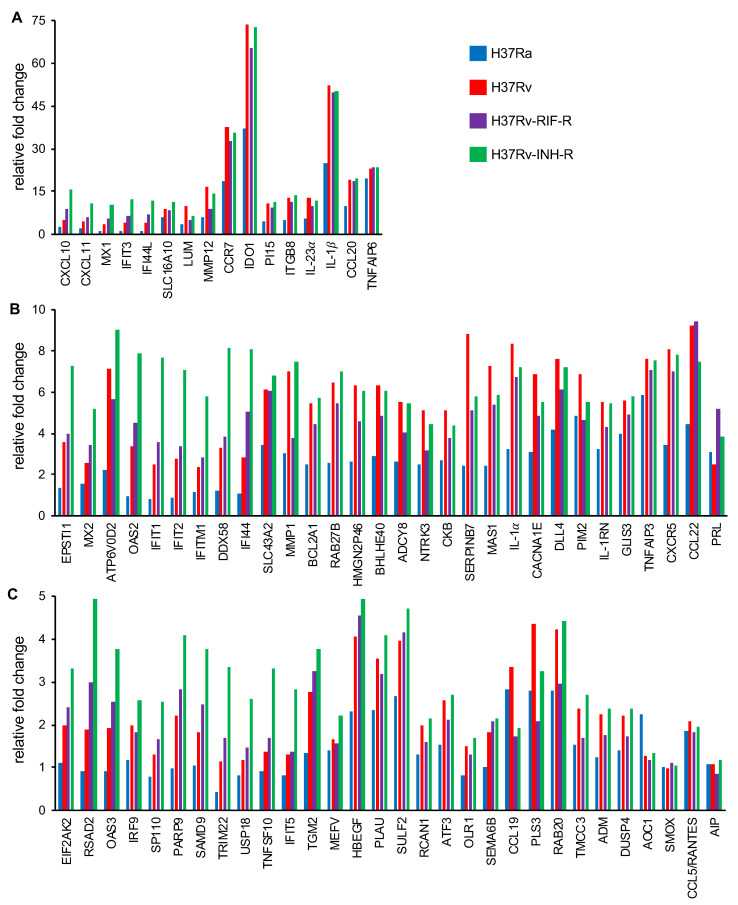
Fold change in the expression of DEGs in THP-1 cell lines infected with reference *M. tuberculosis* strains relative to mock-infected cells (24 h post-infection; *p* < 0.05). Representatives of different degrees of variation in gene expression are depicted in this figure. (**A**) Relative fold changes in gene expression above 10 are shown. (**B**) Relative fold changes in gene expression between 5 and 10 are shown. (**C**) Relative fold changes in gene expression between 1 and 5 are shown.

**Figure 5:**
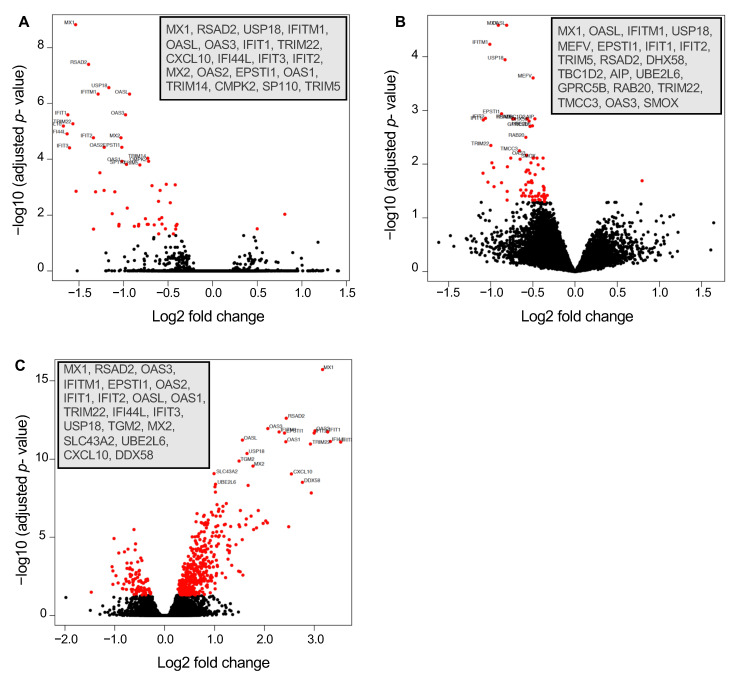
Volcano plots displaying differentially expressed genes in THP-1 cells in three different comparison groups (24 h post-infection; *p* < 0.05). The 20 most highly significant DEGs in each plot are indicated in the insets. These genes all indicate higher expression in H37Rv-INH-R-infected cells, relative to H37Rv- and H37Rv-RIF-R-infected cells. (**A**) H37Rv vs. H37Rv-INH-R. (**B**) H37Rv-RIF-R vs. H37Rv-INH-R. (**C**) H37Rv-INH-R vs. H37Ra.

**Figure 6:**
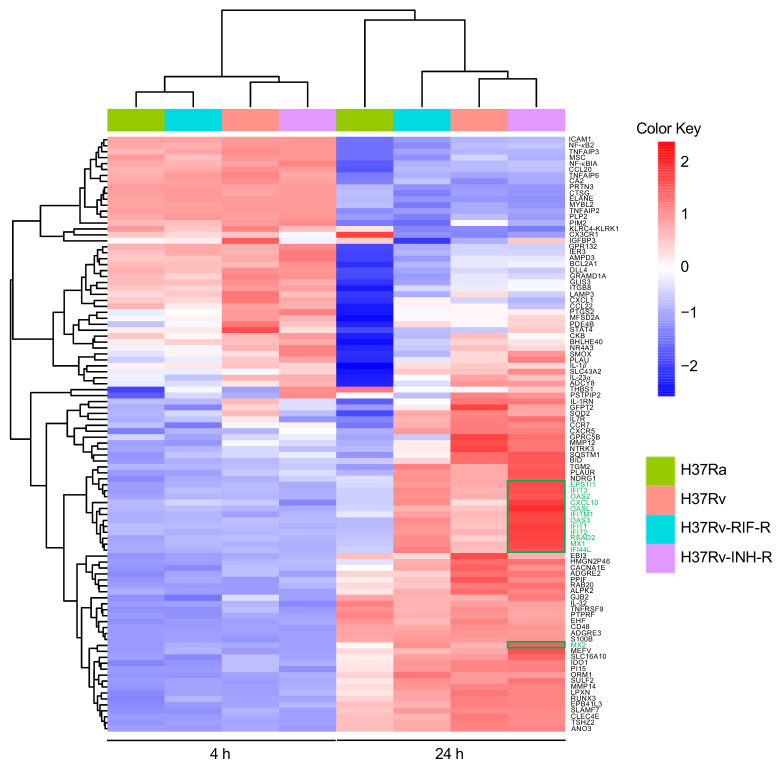
Heatmap and clustering across *M. tuberculosis*-infected THP-1 cells relative to mock-infected cells at 4 h and 24 h post-infection, using the top 100 most significant DEGs in cells infected with isoniazid-resistant H37Rv strain at 24 h post-infection. Samples with a relatively high expression of a given gene are shown in red, and samples with a relatively low expression are shown in blue. Lighter color shades and white indicate genes with intermediate expression levels. Some of the interferon-stimulated genes (ISGs) are marked in green, and their overexpression in cells infected with the isoniazid-resistant H37Rv strain is indicated with a green box in the plot.

Comparison of relative expression of DEGs at 24 h post-infection revealed distinct expression patterns. Whereas some DEGs were up-regulated to comparable levels in all infections (e.g., TNFAIP6), some DEGs, such as IDO1 and IL-1β, were up-regulated to comparable levels in all infections with the virulent reference strains but expressed less in cells infected with the avirulent strain H37Ra (Figure 4A). Strikingly, a subset of DEGs was most strongly induced in H37Rv-INH-R-infected cells, including many interferon-stimulated genes (ISGs), such as CXCL10, MX1, OAS2, IFIT1, DDX58, eIF2aK2 (PKR), RSAD2, and TRIM22 (Figure 4A–C). Relative gene expression differences between infections with H37Rv-INH-R vs. the other strains are also shown in the volcano plots (Figure 5). As a result of this striking differential expression in cells infected with H37Rv-INH-R at 24 h compared to the other infections, we selected the top 100 highly expressed DEGs in H37Rv-INH-R-infected cells vs. mock-infected cells at 24 h and generated a heatmap based on these genes, with all comparison groups, at 4 h and 24 h (Figure 6). This analysis showed that the gene expression patterns varied between infections with the four *M. tuberculosis* strains. Among the DEGs shown in the heatmap, we observed a stronger expression of ISGs associated with H37Rv-INH-R infection (Figure 6).

To determine the key transcription factors that potentially regulate the most significantly expressed DEGs in infected THP-1 cells, we used the interactive web-based program PathwayNet, publicly available at http://pathwaynet.princeton.edu/ (accessed on 22 October 2024) [51]. We examined the interaction predictions for each DEG query in the transcription regulation analysis, and we found the highest relationship confidence in two major transcription factors: signal transducer and activator of transcription 1 (STAT1) and nuclear factor kappa-light-chain-enhancer of activated B cells (NF-κB). Differentially expressed ISGs in H37Rv-INH-R were associated with STAT1 signaling, while the expression of many cytokines, such IL-1β, was associated with NF-κB signaling.

### 3.3. GO and KEGG Enrichment of DEGs in Immune System-Related Pathways

In order to analyze whether DEGs are associated with specific signaling pathways, we performed GO and KEGG enrichment analyses, and we compared two infection groups: drug-sensitive H37Rv vs. H37Rv-INH-R (55 DEGs) and H37Rv-RIF-R vs. H37Rv-INH-R (77 DEGs). Only up-regulated DEGs were considered in these analyses, since only one DEG was down-regulated in the comparison groups.

**Figure 7:**
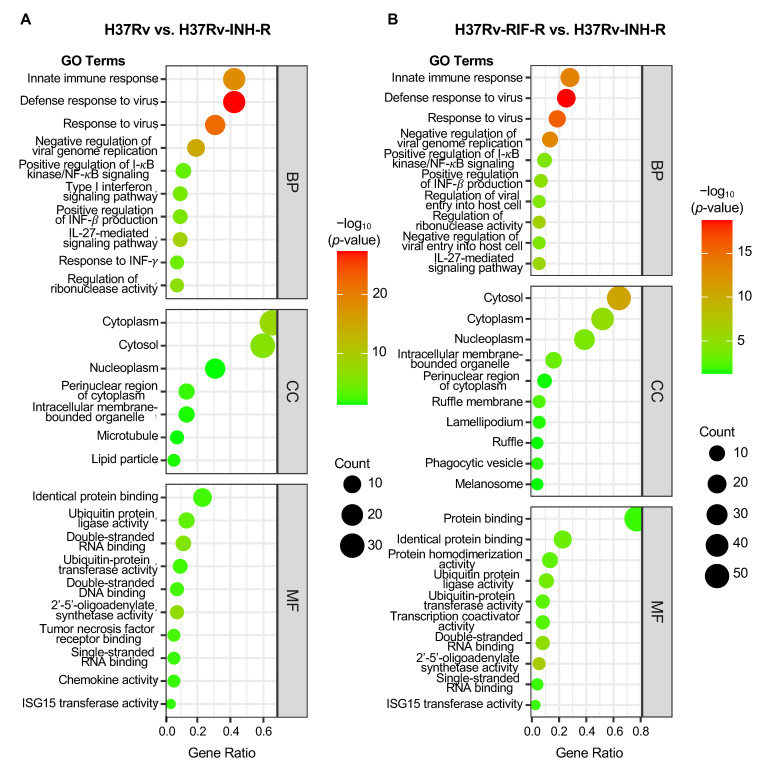
Bubble plot of GO enrichment analysis of up-regulated DEGs in cells infected with the isoniazid-resistant H37Rv strain (24 h post-infection; *p* < 0.05). All GO terms are grouped into three categories: biological process (BP), cellular component (CC), and molecular function (MF). (**A**) Comparison group: drug-sensitive H37Rv vs. isoniazid-resistant H37Rv. (**B**) Comparison group: rifampin-resistant H37Rv vs. isoniazid-resistant H37Rv.

For the GO enrichment analysis, the 10 most significant biological processes, cellular components, and molecular functions identified are shown in Figure 7A,B and in Table 1 and Table 2. For biological processes, the up-regulated DEGs mainly correlated with innate immune response, defense response to virus, and negative regulation of viral genome replication. The up-regulated DEGs were involved in cellular-component terms, mainly including cytoplasm, cytosol, and nucleoplasm. For molecular functions, the up-regulated DEGs were significantly enriched in terms of protein binding, ubiquitin-protein activity, double-stranded RNA binding, and 2′-5′-oligoadenylate synthetase activity.

**Figure 8:**
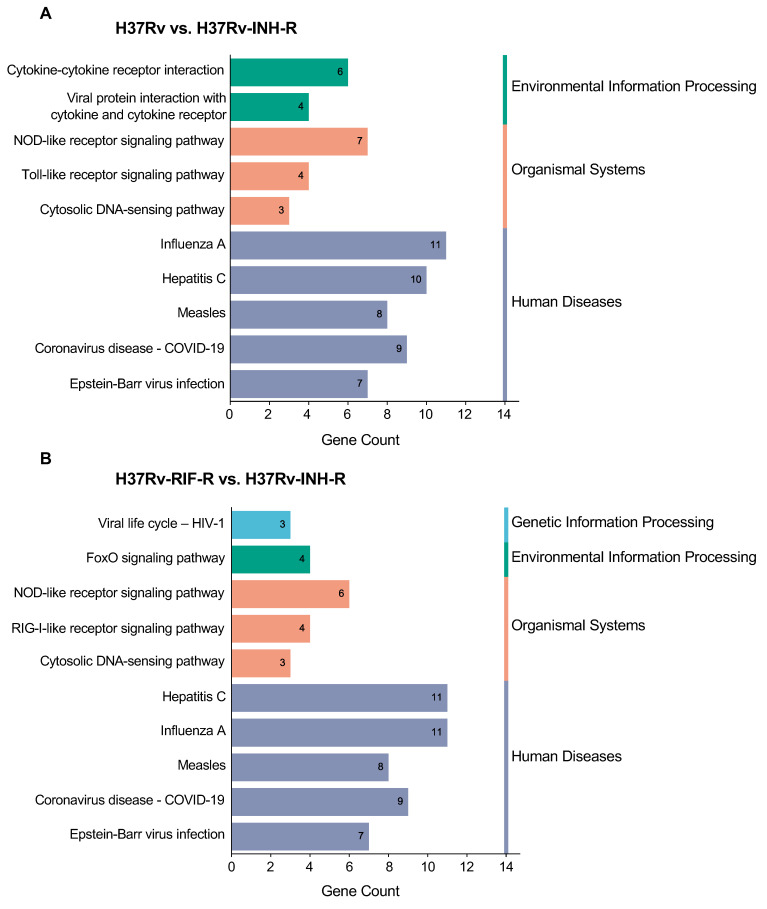
KEGG analysis of up-regulated DEGs in cells infected with the isoniazid-resistant H37Rv strain. The first ten enriched pathways at 24 h post-infection are shown (*p* < 0.05). The KEGG pathways were subsequently divided into three or four categories: environmental information processing, organismal systems, genetic information processing and human diseases. (**A**) Comparison group drug-sensitive H37Rv vs. isoniazid-resistant H37Rv. (**B**) Comparison group rifampin-resistant H37Rv vs. isoniazid-resistant H37Rv.

The top 10 KEGG enrichments correlated with virus and immune system-related pathways, including influenza A, hepatitis C, NOD-like receptor signaling, DNA and RNA sensing, cytokine–cytokine receptor interaction, and FoxO signaling (Figure 8A,B; Table 3 and Table 4).

## 4. Discussion

Our pilot study revealed considerable differences in the host response to infection with different reference *M. tuberculosis* strains. The analysis of overlapping genes indicated distinct profiles in infected cells. While 319 DEGs had a unique profile associated with H37Rv-RIF-R-infected cells, no DEG was found to be unique to the cells infected with the other strains. Moreover, the pairwise comparison between the different *M. tuberculosis*-infected cells indicated 226 unique genes within the cells infected with the drug-sensitive strains (H37Rv vs. H37Ra) but not with the drug-resistant strains. Only a small number of DEGs (55 genes) were also common between all four strains, even though they are all derivatives from the same strain, H37 [52]. This finding emphasizes the complexity of the host immune response to *M. tuberculosis* infection and warrants further investigations that will lead to a better understanding of the mycobacterial strain- and isolate-specific interactions with their host cells.

We observed major differences in host gene expression when comparing infections with the avirulent H37Ra strain and all virulent H37Rv strains. The expression of IDO1 and IL-1β in the infected cells was stronger with all virulent *M. tuberculosis* strains, compared to infections with the avirulent strain. Previous work has shown an up-regulation of IDO1 expression in human and murine macrophages upon infection with reference *M. tuberculosis* strains (e.g., H37Rv), and this finding correlated with higher bacterial burden [53,54]. IL-1β was also shown to be essential in the host resistance to *M. tuberculosis* infection in mouse models [55,56,57]. A comparison of THP-1 cells infected with H37Rv and the non-virulent H37Ra or BCG previously revealed a significant increase in IL-1β and other pro-inflammatory cytokines in H37Rv-infected cells [50]. Differences in macrophage response between clinical latent TB and active TB cases also correlated with variations in IL-1β expression levels [58]. Furthermore, another study suggested a mechanism driven by IL-1β, in which modern *M. tuberculosis* lineages induce more IL-1β expression in comparison with ancient lineages, which might contribute to a higher replication rate of the intracellular bacilli [59]. Clinical isolates of *M. tuberculosis* were also shown to differentially induce IL-1β secretion from macrophages in vitro [60]. In agreement with these findings, our data confirmed differences in the IDO1 and IL-1β expression between four different reference *M. tuberculosis* strains, with the virulent strains showing the highest expression in these genes. Given the role of IDO1 and IL-1β in TB pathogenesis, this suggests therefore that both of these genes could be considered potential biomarkers for TB infection and could lead to an exciting possibility for the development of novel host-directed TB interventions.

**Figure 9:**
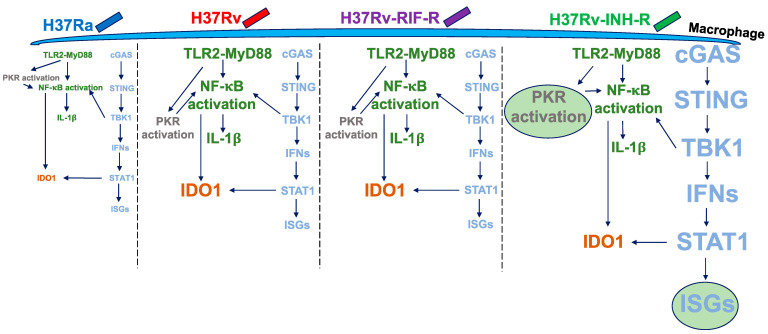
Model for host signaling response to reference *M. tuberculosis* strains. Different font size is used to illustrate the changes in host response to the different reference *M. tuberculosis* strains. Higher gene expression is shown in a big font size, and lower gene expression is shown in small font size. H37Rv-INH-R induced the highest expression of ISGs and PKR compared to the other strains, as indicated by the circles and the big font size.

The most striking result of our study is that the infection with H37Rv-INH-R generated a much higher induction of ISGs in comparison to cells infected with H37Rv and H37Rv-RIF-R. Our data indicate that STAT1 was a major transcription factor associated with the DEGs in response to H37Rv-INH-R infection. Multiple recognition and signaling pathways for the host innate immune responses to *M. tuberculosis* have been documented and characterized, with TLR2-MyD88 and cGAS-STING playing major roles [61]. Our observation that H37Rv-INH-R infection caused the highest ISG expression levels suggests a stronger induction of STAT1 as a result of cGAS-STING activation, rather than TLR2-MyD88 activation. Different signaling pathways may induce changes in the host response to *M. tuberculosis* infection [28]. Based on our findings, we propose a model for the host signaling response to the different reference *M. tuberculosis* strains (Figure 9). In addition to the overexpression of ISGs in the cells infected with the isoniazid-resistant *M. tuberculosis* strain, we also found a higher induction of PKR (eIF2aK2) in those cells. PKR plays a key role in controlling virus infection and has been shown to modulate cytokine expression in response to BCG infection [62] and potentially restrict intracellular replication of the H37Rv-derived strain mc^2^6206 [63]. PKR is also involved in NF-κB activation by more strongly affecting proteins with shorter half-lives, such as the NF-κB inhibitor IκBα [64,65,66]. This links PKR to the observation that NF-κB dynamics play an important role in TB pathogenesis [67,68]. In agreement, we identified NF-κB as the other major transcription factor associated with the DEGs. GO and KEGG pathway enrichment analyses also demonstrated that the up-regulated DEGs in isoniazid-resistant infected cells were significantly enriched in various pathways, especially innate immune response, defense response to viruses, cytoplasm, and protein binding. We found an unexpected up-regulation of many ISGs (e.g., OAS and DDX58) in cells infected with H37Rv-INH-R, and both KEGG and GO analyses indicated an enrichment in the NOD-like receptor signaling pathway, double-stranded RNA binding, and 2′-5′-oligoadenylate synthetase activity. Both PKR and OAS-RNase L pathways have been shown to play an important role in host immune defense and in inducing an increased secretion of IL-1β [69,70,71]. Therefore, it would be interesting to determine how host immunity could be differentially triggered in infections with different *M. tuberculosis* strains through the activation of some of the pathways identified in this pilot study and the overexpression of IL-1β. Our understanding of the differential gene expression in cells infected with different *M. tuberculosis* strains still remains very limited. The *M. tuberculosis* cell wall plays a critical role in modulating the host immune response [22,72]. However, little is known about how its complex structural variations between mycobacterial strains (e.g., drug-sensitive vs. drug-resistant) can influence infection and disease outcome [73,74]. It is therefore important to elucidate how different signaling pathways triggered by the infection with a given *M. tuberculosis* strain, as proposed in our model, could induce ISG and PKR expression after infection with clinical strains, thus improving our understanding of TB pathogenesis and the host immune response to *M. tuberculosis* infection.

The mechanism of INH resistance is complex and has been thoroughly studied in an attempt to improve early diagnosis of INH-resistant *M. tuberculosis* strains [75,76,77,78,79,80,81,82,83]. It is mainly mediated by mutations in the *katG* gene or in the *inhA* regulatory regions [84]. INH is activated by the catalase–peroxidase encoded by *katG*, and this activation interferes with the biosynthesis of mycolic acids by inhibiting NADH-dependent enoyl-ACP reductase encoded by *inhA*. Mutations in other genes have been also associated with INH resistance; however, they are not as common as the ones in *katG* (42 to 95%) and *inhA* (6 to 43%) in *M. tuberculosis* clinical strains, and their mode of action in INH resistance has not been fully elucidated [85]. Nevertheless, not all INH-resistant strains harbor defined genetic mutations or a defined mechanism associated with resistance to this drug, thus complicating efforts to identify those strains [86]. In addition, some studies indicated that some *M. tuberculosis* strains acquire drug resistance at higher rates, suggesting a higher mutation rate in these strains and a higher probability that these strains will develop multidrug resistance [87,88]. Resistance to INH is linked to all multidrug-resistant forms of TB (MDR, XDR, and TDR). Several studies have shown that INH resistance is acquired first, followed by resistance to rifampin and the other anti-TB drugs [81,83,89,90,91], thus highlighting the significant impact of INH on the success of treatment in active and latent TB and warranting further efforts to understand the global burden of INH-resistant TB [92].

Our data showed an overexpression of ISGs in THP-1 cells infected with the INH-resistant strain, H37Rv-INH-R, in which the *katG* gene is deleted. However, it is still unknown how the disruption of *katG* and *inhA*, by mutation or deletion, impacts the host response to INH resistance. It is therefore important to define the role of mutations in these genes in regulating the host immune response to INH-resistant *M. tuberculosis* infection. Such findings will provide a strong foundation for the impact of INH resistance-conferring mutations on the differential host response, which might open up new research avenues to further explore the biological function of these genes and to clarify the underlying molecular mechanisms involved in TB pathogenesis. Nevertheless, further studies are needed to confirm the identified DEGs and pathways in human infections with clinical *M. tuberculosis* strains.

The differential host immune response to different *M. tuberculosis* strains (e.g., overexpression of ISGs in the cells infected with H37Rv-INH-R) provides opportunities to define a new facet of host–pathogen interactions that differentially regulates signaling pathways in response to INH-resistant and other *M. tuberculosis* strains. A recent study investigating a broad profiling of both host and pathogen traits following *M. tuberculosis* infection with H37Rv strain in a genetically diverse mouse panel indicated distinct immune states and variable inter-species genetic interactions that affect TB pathogenesis [93]. A few studies have already suggested the power of gene expression signatures to detect active TB, differentiate between latent and active TB, and even predict TB disease risk and monitor treatment response [94,95,96,97]. Each of these studies has identified a set of gene signatures for TB; however, our study focused, for the first time, on investigating possible gene signatures associated with different *M. tuberculosis* strains. The overexpression of ISGs was found to be unique to cells infected with an isoniazid-resistant *M. tuberculosis* strain, thus warranting further investigation of such a signature in clinical strains or prospective cohort studies. A recent study identified host blood transcriptional signature genes in rifampin-resistant TB infection in comparison with drug-sensitive TB infection [98]. Some DEGs have been identified and were suggested as possible biomarkers to differentiate MDR rifampin-resistant TB patients from drug-sensitive or mono-resistant patients. The characterization of such differential host response has therefore the potential to improve our understanding of the complex and dynamic interactions between *M. tuberculosis* strains and the host.

While we found compelling differences in the host response to different *M. tuberculosis* strains, it is important to acknowledge some limitations that need to be addressed in future studies. Even though THP-1 cells represent a good model to study *M. tuberculosis* interaction with macrophages, the use of other cell lines and ex vivo monocyte-derived macrophages would generate a more thorough analysis of the host immune response to different *M. tuberculosis* strains. Other important follow-up studies can include infection with different clinical *M. tuberculosis* strains, for which the host response can be analyzed by RNA sequencing; quantitative RT-PCR; and the analyses of differential protein expression of differentially regulated gene products such as ISGs, IDO1, and IL-1β.

## 5. Conclusions

Our data showed that each mycobacterial strain was able to induce unique host immune responses in the same cell line, which could have clinical and pathological relevance and lays the groundwork for future studies to investigate strain-specific host–pathogen interactions with clinical *M. tuberculosis* strains and human monocyte-derived macrophages, and ultimately to develop potential new models for host-directed TB therapies. These findings also provide opportunities for new efforts to develop potential biomarkers and/or therapeutic targets for INH resistance and other TB phenotypes.

## Figures and Tables

**Table 1:** Top 10 enriched GO terms of DEGs (H37Rv vs. H37Rv-INH-R).

GO Category	GO Term	Description	Gene Number	*p*-Value	Fold Enrichment	Bonferroni	FDR
BP	GO:0051607	Defense response to virus	22	5.12 × 10^−28^	36.71	1.91 × 10^−25^	1.78 × 10^−25^
	GO:0009615	Response to virus	16	1.03 × 10^−22^	56.31	3.83 × 10^−20^	1.78 × 10^−20^
	GO:0045087	Innate immune response	22	4.35 × 10^−19^	13.8	1.62 × 10^−16^	5.03 × 10^−17^
	GO:0045071	Negative regulation of viral genome replication	10	2.00 × 10^−15^	84.16	7.45 × 10^−13^	1.73 × 10^−13^
	GO:0070106	Interleukin-27-mediated signaling pathway	5	1.26 × 10^−9^	276.53	4.70 × 10^−7^	8.75 × 10^−8^
	GO:0060700	Regulation of ribonuclease activity	4	1.52 × 10^−7^	309.71	5.67 × 10^−5^	8.78 × 10^−6^
	GO:0032728	Positive regulation of interferon-β production	5	3.42 × 10^−6^	47.21	0.0013	1.70 × 10^−4^
	GO:0060337	Type I interferon signaling pathway	5	8.97 × 10^−6^	37.22	0.0033	3.89 × 10^−4^
	GO:0034341	Response to interferon-γ	4	4.27 × 10^−5^	57.35	0.016	0.0016
	GO:0043123	Positive regulation of I-κB kinase/NF-κB signaling	6	1.41 × 10^−4^	11.73	0.051	0.0045
CC	GO:0005737	Cytoplasm	34	1.35 × 10^−8^	2.44	1.08 × 10^−6^	1.08 × 10^−6^
	GO:0005829	Cytosol	31	8.20 × 10^−7^	2.27	6.56 × 10^−5^	3.28 × 10^−5^
	GO:0048471	Perinuclear region of cytoplasm	7	0.0085	3.84	0.494	0.226
	GO:0005811	Lipid particle	3	0.027	11.53	0.887	0.463
	GO:0043231	Intracellular membrane-bounded organelle	7	0.029	2.92	0.904	0.463
	GO:0005874	Microtubule	4	0.044	4.98	0.973	0.517
	GO:0005654	Nucleoplasm	16	0.045	1.63	0.975	0.517
MF	GO:0001730	2′-5′-oligoadenylate synthetase activity	4	6.94 × 10^−8^	370.84	8.88 × 10^−6^	8.26 × 10^−6^
	GO:0003725	Double-stranded RNA binding	6	1.48 × 10^−6^	30.07	1.89 × 10^−4^	8.79 × 10^−5^
	GO:0061630	Ubiquitin-protein ligase activity	7	2.51 × 10^−4^	7.7	0.032	0.01
	GO:0005164	Tumor necrosis factor receptor binding	3	0.0032	34.77	0.339	0.072
	GO:0003690	Double-stranded DNA binding	4	0.0036	12.68	0.374	0.072
	GO:0042802	Identical protein binding	12	0.0041	2.62	0.413	0.072
	GO:0004842	Ubiquitin-protein transferase activity	5	0.0042	7.42	0.421	0.072
	GO:0003727	Single-stranded RNA binding	3	0.0071	23.18	0.601	0.102
	GO:0008009	Chemokine activity	3	0.0077	22.25	0.630	0.102
	GO:0042296	ISG15 transferase activity	2	0.0105	185.42	0.742	0.125

**Table 2:** Top 10 enriched GO terms of DEGs (H37Rv-RIF-R vs. H37Rv-INH-R).

GO Category	GO Term	Description	Gene Number	*p*-Value	Fold Enrichment	Bonferroni	FDR
BP	GO:0051607	Defense response to virus	19	1.73 × 10^−19^	22.33	1.08 × 10^−16^	1.05 × 10^−16^
	GO:0009615	Response to virus	14	1.10 × 10^−16^	34.70	6.91 × 10^−14^	3.32 × 10^−14^
	GO:0045087	Innate immune response	21	3.16 × 10^−14^	9.28	1.97 × 10^−11^	6.36 × 10^−12^
	GO:0045071	Negative regulation of viral genome replication	10	6.14 × 10^−14^	59.27	3.82 × 10^−11^	9.26 × 10^−12^
	GO:0060700	Regulation of ribonuclease activity	4	4.51 × 10^−7^	218.11	2.80 × 10^−4^	5.43 × 10^−5^
	GO:0070106	Interleukin-27-mediated signaling pathway	4	1.57 × 10^−6^	155.79	9.75 × 10^−4^	1.58 × 10^−4^
	GO:0032728	Positive regulation of interferon-β production	5	1.44 × 10^−5^	33.25	0.01	0.001
	GO:0046596	Regulation of viral entry into host cell	4	6.64 × 10^−5^	49.57	0.04	0.005
	GO:0046597	Negative regulation of viral entry into host cell	4	7.62 × 10^−5^	47.41	0.046	0.005
	GO:0043123	Positive regulation of I-κB kinase/NF-κB signaling	7	8.08 × 10^−5^	9.64	0.049	0.005
CC	GO:0005829	Cytosol	48	1.98 × 10^−11^	2.42	2.78 × 10^−9^	2.76 × 10^−9^
	GO:0005737	Cytoplasm	39	6.74 × 10^−6^	1.93	9.43 × 10^−4^	4.68 × 10^−4^
	GO:0005654	Nucleoplasm	29	1.13 × 10^−4^	2.04	0.016	0.005
	GO:0043231	Intracellular membrane-bounded organelle	12	5.72 × 10^−4^	3.45	0.077	0.02
	GO:0032587	Ruffle membrane	4	0.0056	11.02	0.542	0.154
	GO:0045335	Phagocytic vesicle	3	0.0256	11.92	0.974	0.594
	GO:0030027	Lamellipodium	4	0.0308	5.80	0.987	0.613
	GO:0048471	Perinuclear region of cytoplasm	7	0.0460	2.65	0.997	0.677
	GO:0001726	Ruffle	3	0.0491	8.35	0.999	0.677
	GO:0042470	Melanosome	3	0.0527	8.02	0.999	0.677
MF	GO:0001730	2′-5′-oligoadenylate synthetase activity	4	2.11 × 10^−7^	259.08	3.86 × 10^−5^	3.78 × 10^−5^
	GO:0003725	Double-stranded RNA binding	6	9.12 × 10^−6^	21.01	0.002	8.16 × 10^−4^
	GO:0061630	Ubiquitin-protein ligase activity	8	2.92 × 10^−4^	6.15	0.052	0.017
	GO:0042802	Identical protein binding	17	5.37 × 10^−4^	2.59	0.093	0.024
	GO:0042803	Protein homodimerization activity	10	0.0017	3.56	0.27	0.061
	GO:0004842	Ubiquitin-protein transferase activity	6	0.0026	6.22	0.3835	0.079
	GO:0003713	Transcription coactivator activity	6	0.0034	5.87	0.4627	0.087
	GO:0003727	Single-stranded RNA binding	3	0.0144	16.19	0.9296	0.271
	GO:0005515	Protein binding	58	0.0146	1.19	0.9325	0.271
	GO:0042296	ISG15 transferase activity	2	0.0151	129.54	0.9387	0.271

**Table 3:** Top 10 enriched KEGG pathway terms of DEGs (H37Rv vs. H37Rv-INH-R).

KEGG Term	Description	Gene Number	*p* Value	Fold Enrichment	Bonferroni	FDR
hsa05164	Influenza A	11	3.61 × 10^−10^	16.49	2.89 × 10^−8^	2.64 × 10^−8^
hsa05160	Hepatitis C	10	3.85 × 10^−9^	16.33	3.08 × 10^−7^	1.40 × 10^−7^
hsa05162	Measles	8	6.44 × 10^−7^	14.76	5.16 × 10^−5^	1.57 × 10^−5^
hsa05171	Coronavirus disease—COVID-19	9	1.63 × 10^−6^	9.95	1.30 × 10^−4^	2.97 × 10^−5^
hsa04621	NOD-like receptor signaling pathway	7	5.41 × 10^−5^	9.75	0.004	7.90 × 10^−4^
hsa05169	Epstein–Barr virus infection	7	9.10 × 10^−5^	8.88	0.007	0.001
hsa04060	Cytokine–cytokine receptor interaction	6	0.0046	5.21	0.307	0.048
hsa04061	Viral protein interaction with cytokine and cytokine receptor	4	0.0062	10.26	0.390	0.056
hsa04620	Toll-like receptor signaling pathway	4	0.0069	9.86	0.424	0.056
hsa04623	Cytosolic DNA-sensing pathway	3	0.0234	12.21	0.849	0.155

**Table 4:** Top 10 enriched KEGG pathway terms of DEGs (H37Rv-RIF-R vs. H37Rv-INH-R).

KEGG Term	Description	Gene Number	*p* Value	Fold Enrichment	Bonferroni	FDR
hsa05160	Hepatitis C	11	5.50 × 10^−9^	13.06	6.88 × 10^−7^	6.72 × 10^−7^
hsa05164	Influenza A	11	1.26 × 10^−8^	11.99	1.57 × 10^−6^	7.67 × 10^−7^
hsa05162	Measles	8	6.67 × 10^−6^	10.73	8.33 × 10^−4^	2.71 × 10^−4^
hsa05171	Coronavirus disease—COVID-19	9	2.23 × 10^−5^	7.23	0.003	6.81 × 10^−4^
hsa05169	Epstein–Barr virus infection	7	5.89 × 10^−4^	6.46	0.071	0.014
hsa04621	NOD-like receptor signaling pathway	6	0.0026	6.08	0.277	0.053
hsa04622	RIG-I-like receptor signaling pathway	4	0.0057	10.66	0.514	0.100
hsa04068	FoxO signaling pathway	4	0.0309	5.69	0.980	0.471
hsa03250	Viral life cycle—HIV-1	3	0.0428	8.88	0.996	0.523
hsa04623	Cytosolic DNA-sensing pathway	3	0.0428	8.88	0.996	0.523

## Data Availability

The complete dataset for this study is available from the NCBI Gene Expression Omnibus database (GEO; https://www.ncbi.nlm.nih.gov/geo/; accessed on 22 October 2024) and is publicly accessed through GEO Series accession number GSE246736.
